# Integrating Wireless Remote Sensing and Sensors for Monitoring Pesticide Pollution in Surface and Groundwater

**DOI:** 10.3390/s24103191

**Published:** 2024-05-17

**Authors:** Titus Mutunga, Sinan Sinanovic, Colin S. Harrison

**Affiliations:** School of Engineering and Built Environment, Glasgow Caledonian University, Glasgow G4 0BA, Scotland, UK; sinan.sinanovic@gcu.ac.uk (S.S.); colin.harrison@gcu.ac.uk (C.S.H.)

**Keywords:** water, pesticide, pesticide detection, wireless sensor, internet of things, big data, machine learning, pollution

## Abstract

Water constitutes an indispensable resource crucial for the sustenance of humanity, as it plays an integral role in various sectors such as agriculture, industrial processes, and domestic consumption. Even though water covers 71% of the global land surface, governments have been grappling with the challenge of ensuring the provision of safe water for domestic use. A contributing factor to this situation is the persistent contamination of available water sources rendering them unfit for human consumption. A common contaminant, pesticides are not frequently tested for despite their serious effects on biodiversity. Pesticide determination in water quality assessment is a challenging task because the procedures involved in the extraction and detection are complex. This reduces their popularity in many monitoring campaigns despite their harmful effects. If the existing methods of pesticide analysis are adapted by leveraging new technologies, then information concerning their presence in water ecosystems can be exposed. Furthermore, beyond the advantages conferred by the integration of wireless sensor networks (WSNs), the Internet of Things (IoT), Machine Learning (ML), and big data analytics, a notable outcome is the attainment of a heightened degree of granularity in the information of water ecosystems. This paper discusses methods of pesticide detection in water, emphasizing the possible use of electrochemical sensors, biosensors, and paper-based sensors in wireless sensing. It also explores the application of WSNs in water, the IoT, computing models, ML, and big data analytics, and their potential for integration as technologies useful for pesticide monitoring in water.

## 1. Introduction

Pesticides play an important role in the agricultural domain, serving as tools for farmers to enhance crop productivity and protect them from pest-induced losses as explained by [1]. Nevertheless, proper measures must be implemented to mitigate potential ecological effects associated with their introduction into the environment. Given the main objective of their application is to eliminate specific life forms, it is imperative to systematically monitor and assess their consequential impacts on flora and fauna to ensure their proper utilization without compromising the delicate balance of ecosystems.

**Figure 1:**
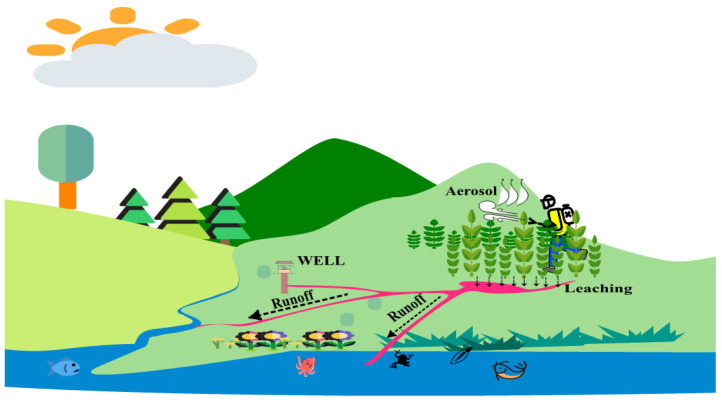
Ways in which pesticides spread into the environment.

Pesticides are mobile because they drift to new regions in the form of aerosols, through surface runoff, leaching, and infiltration processes, to reach waterways and cause contamination as illustrated in Figure 1. Independent studies conducted in different parts of the world have shown pesticides have been detected in surface and groundwater [2,3]. Researchers in Germany found that of the total sites selected for monitoring, 1/3 contained active ingredients [4]. A similar study revealed the presence of glyphosate and its degradation component AMPA in Italy. According to a report by the Heinrich Böll Foundation [4], the lack of routine monitoring regimes in countries poses a significant risk to public health, particularly concerning pesticide residues in water. The governments of developing countries confront substantial hurdles in regulating the lawful trade of pesticides. These challenges are compounded by the proliferation of counterfeit imports and illicit practices among local traders, who clandestinely market their own pesticide variants to cater for small-scale farmers seeking affordable options.

In recent years, the illicit pesticide trade has witnessed a sharp increase, fuelled by enticing profit margins and inadequate government oversight. Simultaneously, the sale of counterfeit pesticides has become a lucrative venture. Notably, in the first four months of 2020, illegal pesticides worth up to 94 million euros were confiscated across the EU and six other countries, including Colombia, Switzerland, and the USA [4].

A significant portion of pesticides used globally comprises Highly Hazardous Pesticides (HHPs), characterized by their elevated threat to human health and the environment. HHPs encompass ingredients deemed carcinogenic, fertility impairing, or gene mutating, or which are listed in internationally binding conventions such as the Montreal, Rotterdam, or Stockholm Conventions on persistent organic pollutants.

Despite stringent regulations within the EU, it remains the world’s largest exporter of pesticides, with a growing focus on investments in the Global South [4]. This trend enables EU-based pesticide companies to export products banned domestically due to their adverse effects, leveraging less stringent regulatory environments in developing countries.

Glyphosate, for instance, has been linked to cancer in humans according to the International Agency for Research on Cancer (IARC) [5]. Studies have also indicated that it interferes with some cells in bees, impairing their learning abilities and navigation [6]. Women and girls are more vulnerable to pesticides as their bodies are more hormonally sensitive [7]. Having a large proportion of fatty tissue makes them store pollutants which tend to bioaccumulate. Studies have discovered traces of pesticides that bioaccumulate in food chains in the breasts of women cancer patients [2,4]. Water contaminated with pesticides is known to cause a range of other illnesses like asthma, dermatitis, and Parkinson’s disease, as well as vomiting, coma, and even death [8,9]. The general impact on biodiversity is devastating as some pesticides like acetamiprid harm non-target insects like pollinators heavily impacting plant biodiversity in natural vegetation. The natural balance of organisms is also affected as some insects control other pests and vectors for human or animal diseases, and their extinction leads to an outbreak of crop pests causing food insecurities. Some insects that act as good sources of proteins and minerals for humans are collected from the wild already contaminated by pesticides [10]. With a growing world population, water continues to be a scarce commodity as demand for it increases across different sectors, including agriculture, domestic use, and industries. Reports have shown that between 2.2 billion and 3.2 billion people lived under water stress for at least one month per year in 2010, and with an annual water demand increase of 1%, it is projected that between 1.7 billion and 2.4 billion people (approximately 1/2 of the global urban population) will face water scarcity in 2050 [11]. Climate change has escalated the situation by altering rainfall patterns and increasing the frequency of hydro-climatic extremes like drought, heat waves, and floods [12]. In addition to the evaluation of water scarcity, which predominantly centres on quantitative considerations, the assessment of water quality assumes a paramount significance as it encompasses its utilization for specific activities such as cooking, drinking, and bathing. Monitoring of pesticide residues has gained a great deal of interest as contamination of water sources continues to increase due to anthropogenic activities.

Studies have been conducted on pesticide detection using various methods [13,14,15,16]. Some must be primarily conducted inside a laboratory due to the complexity of the procedures involved, while other methods permit users to carry out their tests in the field. Studies have indicated laboratory tests, commonly referred to as traditional methods, are time-consuming, laborious, and result in delays. The field-deployable methods are still at the testing stage and have not reached commercialization, while those that have are not scalable to allow wide coverage. Some of the key highlights of the papers surveyed indicate the following.

Plug-and-play sensors are currently non-existent. The challenge arises from the nature of most pesticides in the market, which predominantly consist of organic compounds. Detecting these compounds poses difficulties as they often do not induce noticeable changes in physicochemical parameters that can be easily detected by existing sensors. Numerous studies dedicated to monitoring water quality indexes within aquatic ecosystems predominantly emphasize physical parameters, often overlooking pesticide contamination. The absence of readily available plug-and-play sensors specifically designed for detecting pesticides poses a significant barrier to the establishment of sensor networks tailored for pesticide monitoring. Given the preference for wireless networks in monitoring scenarios, the unavailability of such sensors further inhibits the development of comprehensive monitoring systems for pesticide contamination in water. A key merit of wireless sensors is that it takes a shorter time to gather data compared to instrumental methods, an approach providing timely and consistent determination of water quality parameters. Significant attention has been placed on WSNs as a result of their suitability and range of advantages. The technology exhibits robustness, reliability, speed, autonomy, and cost-effectiveness [17]. WSNs promote access to spatiotemporal data by being deployed over large geographical areas, giving a picture of relative levels of contamination. Due to their small size and ability to withstand harsh conditions, they enable remote monitoring in logistically hard-to-reach areas like valuable water bodies or dense agricultural areas [18].

Current pesticide testing relies on centralized systems at storage points before water distribution, often employing sampling methods due to cost constraints. Analytical methods of determination are used which involve carrying samples to testing stations. Reliance on periodic sampling regimes can easily miss important events like short-term spikes in pollution. These procedures are intensive in terms of manual labour requirements and lab analysis sessions, making them expensive affairs to undertake and not cost-effective for detection from many sources.

Regions that do not have a piped water infrastructure and depend on distributed water sources for their domestic needs create a challenge for centralized systems of testing. Decentralized efforts are essential for overcoming the limitations observed in centralized approaches. The incorporation of newer technologies like wireless sensor networks (WSNs) and the Internet of Things (IoT) give some promising results in terms of access and efficiency of these solutions. While WSNs are dedicated to sensing and transmission of data, the IoT goes beyond to incorporate more complex tasks like decision-making, automation, and control. IoT-enabled devices provide real-time data to the users with access to the networks by either using notification messaging or continuous streaming of data by repeatedly updating the portals. In addition, the IoT enables remote monitoring, is scalable, and lacks physical connections [19].

The mobility of pesticides through various processes underscores the importance of frequent routine monitoring and surveillance to identify and mitigate potential risks effectively. This calls for bespoke solutions with low-cost-detection and easily accessible methods. Current methods of testing are either too complex, with bulky equipment or prototype devices that are still undergoing lab development. Commercial point-of-care testing solutions are prohibitively expensive and limited to detecting samples in spiked pure water, an unrealistic scenario in complex environmental settings with myriad compounds. Additionally, existing alternatives are often localized and lack connectivity. Leveraging emerging technologies offers promising avenues for developing devices with human interfaces for use in local communities. The use of friendly devices fosters community engagement, promoting environmental stewardship.

Certain countries use proxy data from pesticide sales as a way of determining pesticide contamination levels. No information is available on their application or their effect on biodiversity. Particularly in numerous African nations, detailed statistics pertaining to pesticide usage per crop are lacking. This deficiency in systematic data collection necessitates the utilization of sales volumes as a surrogate measure for estimating pesticide utilization [4]. Big data analytics combined with machine learning can identify anomalous patterns in sales data, indicating potential misuse and overuse, and unusual spikes or trends can trigger investigations. Machine learning algorithms can be trained using data from pesticide sales and other diverse data sets, including land use or weather patterns. These models can forecast contamination levels across different regions. Nevertheless, the precision of these predictions is anchored on the quality of training data and the choice of models [20]. ML techniques can play a role in data refinement, rectifying errors and pinpointing biases or variances and thereby improving the data quality. Furthermore, integrating ML and big data analytics can enhance the computing power of sensors for pesticide detection.

Various methods of pesticide detection are analysed in the following sections, giving more emphasis to those that are low-cost for large-scale applications and can easily be integrated into a network for wider access. Wireless networks are discussed as potential connectivity links for transmitting test results from the field due to their several advantages. In the discourse surrounding the refinement of detection methodologies, ML and big data represent a compelling frontier in the advancement of accurate detections. By harnessing their potential, future research endeavours hold the promise of yielding refined and robust detection endowed with heightened accuracy and efficiency.

Our research sought to explore the existing literature regarding wireless sensor networks (WSNs) utilized for the detection of pesticides in water. Despite our thorough investigation, it became evident that comprehensive reviews specifically addressing the integration of WSNs for pesticide detection in aquatic environments are notably scarce. While there exists a body of literature offering comprehensive reviews on WSNs employed for monitoring water quality [21,22,23,24], these reviews predominantly lack a focused examination of pesticide detection.

Moreover, despite the explosion of studies dedicated to the Internet of Things (IoT)-based water monitoring systems [25,26,27,28,29], the literature encompassing reviews of IoT pesticide monitoring in water remains conspicuously limited. Our review of the available literature highlighted a notable deficiency in this specific domain.

Nevertheless, we encountered several insightful surveys concentrating on point-of-care testing methodologies for pesticide detection in fruits, vegetables, and foods [30,31,32,33,34,35]. Notably, a subset of these surveys selectively explores the analysis of pesticide detection within aquatic environments [36,37,38].

In this paper, we begin by presenting analytical methods for detecting pesticides in water. We examined the rapid detection methods detailing various modifications undertaken to enhance their suitability for point-of-care testing by shortening detection time. In Section 3, we present an assessment of IoT as applied to pesticide sensing, while Section 4 focuses on WSNs in water quality monitoring. Section 5 and Section 6 are high-level approaches with significant potential for widespread application in the realm of aquatic pesticide testing, demonstrating considerable promise for advancing in the field. Finally, we summarize our work and give avenues for future research.

## 2. Methods of Pesticide Detection

### 2.1. Instrumental Techniques

Instrumental techniques such as gas chromatography (GC) and liquid chromatography (LC) coupled to a favourable detector for pesticide detection require several steps for sample preparation, namely separation, pre-concentration, then detection and analysis [39,40]. The popularity of instrumental techniques in the detection of pesticides in water lies in their superiority in performing trace analysis in complex matrices [41]. Very low limits of detection (LOD) and limits of quantification (LOQ) are achieved when these techniques are coupled with tandem mass spectrometry (MS/MS), a procedure commonly applied while screening a wide variety of unknown contaminants in non-target analysis [42,43]. A study carried out in France applying online solid phase extraction ultra-power liquid chromatography-tandem mass spectrometry (SPE-UPLC-MS/MS) to herbicides and pharmaceutical compounds identified 15 emerging micropollutants in water at low detection and quantification limits averaging 10 ng per litre and 30 ng per litre, respectively [44]. However, research points to some key challenges for their applicability in rapid detections required in point-of-care testing of pesticides. Instrumental techniques are laborious and consume significant volumes of organic solvents, and their complexity only permits them to be performed in specialized labs [45,46,47].

### 2.2. Spectroscopic Techniques

The interaction of light (optical spectroscopy) or laser (Raman spectroscopy) with a sample provides details about its electronic structure, which are important for the identification of substances [48]. Optical spectroscopy measures the absorption, emission, or reflection of a sample by directing light in the ultra-violet visible spectrum infra-red (UV-VIS-NIR) regions through or off it [49]. The use of this approach in the determination of pesticides in water is limited since water shows broadband absorption and overlapping spectra, demanding multivariate techniques to extract information [50]. The use of fluorescent quenching is used in the design of biosensors where fluorescent dyes, quantum dots (QDs), carbon dots (CDs), and metallic nanoparticles (NPs) are used as probes in detecting organophosphorus pesticides using the enzyme inhibition method [51]. Neither Raman nor Surface-enhanced Raman (SERs) produce high intensities for vibrational modes of water molecules, an aspect utilized in the detection of water pollutants [52]. SERs have been widely studied due to the development of nano-substrates such as semi-conductor NPs, quantum dots, and graphene dots, in addition to the noble metal NPs [53]. Even though spectroscopic techniques are non-destructive and environmentally friendly as indicated in Table 1, their major challenges in pesticide detection are their expensive and complex instrumentation [54].

### 2.3. Emerging Sensor Technologies

#### 2.3.1. Electrochemical Sensors

Electrochemical sensors work by adsorbing the analyte of interest on the surface of the electrode, followed by a redox reaction. A quantitative relationship is established between the electrical signals produced and the analyte concentration [55]. Identification of different analytes involves the observation of oxidized or reduced peaks of the voltammograms [14]. Electrochemical cells usually consist of 3 electrodes: a working electrode, a counter electrode, and a reference electrode. A reaction of interest takes place on the working electrode, and therefore a great deal of current research is focussed on improving it. Modifications are performed on the electrodes to improve their efficiency [16]. This will involve etching techniques to enhance surface roughness for the adsorption of pollutants or the introduction of active materials like carbon nanomaterials, quantum dots, conductive organics, metal oxide nanoparticles, antibodies, and enzymes [56]. Additionally, modifying the surfaces using nano-structured materials promotes faster electron transfer kinetics, resulting in quicker response times ideal for wireless sensing. The electrical characterization techniques employed are differential pulse voltammetry (DPV), square wave voltammetry (SWV), chronoamperometry, and electrical impedance spectroscopy (EIS) [57]. The production of an electrical signal output requires fewer signal processing stages before it is used in detection activities, which is an advantage for rapid monitoring schemes. Faster response times are also observed by the use of signal amplification, such as enzyme or catalytic reactions. This makes electrochemical sensors a good option to use when speed of sensing is a requirement, as demanded in wireless sensing. Researchers have noted electrochemical sensing confers several advantages, including low cost, simplicity, precision, sensitivity, and suitability for point of care [58]. However, their commercial viability, inability to detect multiple analytes, and failure to give results at high analyte concentrations remain their main disadvantages [57].

#### 2.3.2. Biosensors

A biosensor is a device that utilizes a biological element for its sensing mechanism. The biological element used interacts with the analyte, and the result is measured and quantified [59]. Some of the biological elements that have been used in the design of sensors are cells, antibodies, enzymes, molecules, and aptamers [60]. Some artificially synthesized ligands called molecularly imprinted polymers have also been utilized in the design of biosensors. Employing techniques for the immobilization of bio-recognition elements like encapsulation or covalent bonding fixes the elements onto the sensor surface, improving stability and thereby reducing response times. Research in biosensors has been fuelled by their features, which include fast detection, high sensitivity, in situ detection, low cost, and miniaturization [61]. The shrinking of biosensors and their integration with microfluidic systems reduces the diffusion distance between the analyte and the recognition elements. This permits quicker transport and mixing, making them suitable for wireless sensor networks. A suitable transduction mechanism transforms the interaction into some measurable quantity. When those components come into contact with analytes, their interaction can result in heat generation which is used for quantification. Other interactions result in a colour change, or the reaction can take the place of a metal surface causing oxidation or reduction. It is also possible to mix the interacting species with some fluorescent elements [15]. A reaction will lead to a decrease in fluorescence, measured using lab spectrometers. Optical and electrochemical enhancements utilizing advanced techniques like surface plasmon resonance (SPR), SERs, or electrochemical impedance spectroscopy (EIS) provide the rapid outputs required in wireless sensing. It is always good to make a good choice of the transduction mechanism to use for a particular reacting species of biomolecules.

(a) Immunosensors

The biological element used in the construction of an immunosensor is an antibody. Antibodies are produced as a result of the immune response of organisms. Antibodies are very specific to the antigens that trigger their production [62]. Immune sensors are classified as labelled and label-free. Non-labelled sensors measure physical changes and kinetic information during Antigen-Antibody (Ag-Ab) immune complex formation. They are mostly applied in the qualitative sensing of Ag via direct observation of signal changes. Labelled sensors use signal-producing labels like enzymes, fluorescent dyes, NPs, and QDs for more sensitive detection in the immune complex [63]. Various studies have been conducted on the detection of pesticides in environmental waters by the binding action of antibodies on pesticide molecules.

Vaid et al. [64] developed immunoprobes by modifying the surface of gold working screen-printed electrodes (SPE). Thiourea was used to immobilize the atrazine-specific antibody on the electrode. In one procedure, thiourea was drop-cast, and then after 10 min of incubation, gold nanoparticles (AUNPs) were added. In the other preparation procedure, AUNPs were linked to thiourea and then immobilized on the SPE. When nanoprobes were tested against glyphosate and bifenthrin, high selectivity was noted. LODs of atrazine were in the range of 0.08–0.06 ng/L. Moreover, the developed immunosensor had a wider linear range of 50 ng/L–30 μg/L and a short response time of 0.5 min.

Fernández et al. [65] applied direct competitive immunoassay on modified screen-printed carbon electrodes. A total of 10 μL of monoclonal imidacloprid antibody was dropped on the surface of the AUNP-modified working electrode and incubated at 4 °C overnight. The surface was washed, and then 1% Bovine serum albumin fraction was added to avoid unspecific absorptions. The limit of detection for imidacloprid was 22 pmol L^−1^, which was below the maximum permissible levels. In addition, they reported the biosensor to have a good accuracy and wider linear range. Other attributes included being faster, cheaper, and simpler.

In their study, Campanile et al. [66] utilized gold-coated magnetic NPs to enhance micro-stirring. Magnetic stirring of NPs is known to improve the sensitivity of the assay by reducing passive diffusion and enhancing their binding efficiency. A photochemical immobilization technique was used to immobilize the antibodies. This was achieved by exposing the antibody solution to ultra-violet radiation. When the Fe_3_O_4_@AuNPs biosensor was tested for glyphosate-spiked tap water, an LOD of 20 ngL^−1^ was obtained. However, the biosensor suffered from the prozone (hook effect), like all the other biosensors produced by the immunoprecipitation scheme.

(b) Enzyme biosensors

The principle of operation is based on the relationship between the toxicity of a pesticide and the decrease in enzyme activity. Quantitative measurement of enzymatic activity is performed before and after exposure to a particular analyte [67]. After exposure to a certain pesticide, this activity is measured by an appropriate transduction technique, e.g., amperometry, spectrometry, fluorometry, potentiometry, etc., depending on the products of the activity. The most commonly used enzymes are Acetylcholinesterase (AChE) and Butyrylcholinesterase (BChE). An important step in the development of enzyme biosensors is the immobilization of the enzyme so that it does not lose function [68]. Enzyme biosensors offer simpler and smoother operation, giving results faster and with higher sensitivity than instrumental techniques.

Zambrano et al. [69] fabricated a horseradish peroxidase enzyme-based biosensor for the detection of glyphosate. The surface of a pencil graphite electrode was cut and washed. Poly sulfone and multiwalled carbon nanotubes were dripped on the cut surface and then immersed in horseradish peroxidase enzyme to produce HRP/PSF/MWCNT/PGE biosensor. Amperometry response showed an LOD and LOQ of 0.225 mgL^−1^ and 0.0084 mgL^−1^, respectively. These limits showed suitability for application in the environmental monitoring of glyphosate as the EPA value is set at 0.7 mgL^−1^ for drinking water. A wide linear range was noted from 0.1 to 10 mgL^−1^. The study further noted the affordability of materials, simplicity, and ease of manipulation as strengths. Catalytic activity decreased from the 11th day onwards, while high selectivity was noted when interfering chemicals were used.

Tun et al. [70] developed an AChE-based biosensor for detecting chlorpyrifos. Surface modification of SPCE was performed using cellulose nanofibers and graphene oxide. Electrochemical reactions were characterized by cyclic voltammetry. The activity of the enzyme decreased as pH deviated from 7. It was further observed that no extra inhibition by chlorpyrifos occurred after 15 min. The study revealed LOD and LOQ as 2.2 nM and 73 nM, respectively. The sensor was found to be stable after 21 days, and no significant interference was noted with other analytes.

(c) Molecular imprinted polymers

Molecular imprinting is the process of creating an impression using a template. This template is an analyte molecule. Once the template is eluted at the end of the process a cavity is left which has affinity for the target molecule. Molecular imprinted polymers (MIP) are artificial receptors that are used to bind target molecules and thus act as recognition elements [71]. Some functional monomers and cross-linking monomers are copolymerized in the presence of a desired target molecule. The method gives freedom to tune the properties of MIPs and even experiment with new designs for which natural antibodies do not exist. Molecular imprinting overcomes some of the limitations noted with other biosensors, like the high cost of antibodies, instability of enzymes, or a strong binding that prevents their re-use [72]. When applied as synthetic recognizers, MIPs have been noted to exhibit some admirable qualities like chemical inertness, stability, and insolubility in organic compounds and water.

Elshafey et al. [73] developed a molecularly imprinted poly(o-pd-co-pyrrole) film on a glassy carbon electrode modified by electrochemically reduced graphene oxide for detecting propachlor in spiked lake water. Modification of the GO/GCE electrode was achieved by sweeping a potential between 0.2 and −1.2 V. Synthesis of the MIP was then followed by dipping the electrode in pyrrole, o-phenylene diamine, and propachlor. There was an observed change in the binding of propachlor to the constructed electrode. The group noted that graphene oxide increased the density of cavities on the film. Sensor values were proportional in the range of 0.1 pM to 0.1 µM and an LOD was observed to be 0.08 pM.

Sihua Peng et al. [74] investigated imidacloprid’s presence in the environment using a molecular imprinted polymer. Graphene oxide was used as a modifier for the SPCE. HAuCl4 deposition solution was applied dropwise and dried, then imidacloprid chitosan stock solution was added at −1 V for 300 s to polymerize the electrode. Differential pulse analysis showed good linear response current with pesticide concentration. Resistance to interference was indicated to be less than 5% for the interfering molecules considered. The group reported an LOD of 0.5 µM; however, they used spiked water samples.

(d) Aptamers

Nucleic acid aptamers are short single-stranded Deoxyribonucleic acid (DNA) or Ribonucleic acid (RNA) molecules that can bind to target molecules with high specificity. These synthetic ligands have a level of selectivity which can be likened to that of antibodies. They are selected from a large library of oligonucleotides through a process called Sequential Evolution of Ligands by Exponential Enrichment (SELEX) [75]. In their selection, copious oligonucleotides are allowed to bind to a certain analyte. Those that bind are eluted and enriched through Polymerase chain reaction (PCR) amplification, and then analytical methods of selection of the best aptamer are performed. Studies have shown that they have high thermal stabilities and longer lifetimes, and can be easily chemically synthesized or modified [76].

Kamkurua et al. [77] investigated the detection of paraquat by aptamer functionalized gold NPs in a SERS substrate. The aptamer was immobilized on SERs chips by dipping them in PQ77-SH aptamer solution for 30 min. The modified chips were then dipped in paraquat and then rinsed in phosphate-buffered saline to remove excess chemicals. Raman measurements were then carried out on the dried samples and spectra were analysed using the hyperspace R package. Signal observations indicated that paraquat signature peaks were broadened and shifted after bonding with the aptamers. A good linear range was obtained between 0.25 and 2.5 μM, and an LOD of 0.10 ± 0.03 μM was recorded. The aptasensor showed good reproducibility with 4% variations of peak intensities and good stability throughout 10 days. It had a good selectivity when detecting paraquat in interfering chemicals like glyphosate and deltamethrin.

Talari et al. [78] studied the detection of diazinon in water using reduced graphene quantum dots (rGQDs) and a multiwalled carbon nanotubes (MWCNTs) aptasensor. The rGQDs were synthesized from citric acid pyrolysis and reacting sodium borohydride (NaBH4) with tetrahydrofuran. To activate the carboxylic groups, EDC and NHS were added and magnetically stirred. The standard aptamer solution (DF20) was then added, followed by MWCNTs. Diazinon measurement was conducted by the fluorescence of the rGQDs-Aptamer-MWCNTs aptasensor at 460 nm. The optimum PH for diazinon was found to be 3–8. The introduction of other pesticides and ions in the matrix did not interfere with diazinon detection of the aptamer, recorded at 3% relative standard deviation (RSD). Linearity was observed in the range of 4–31 nM with an LOD of 0.4 nM.

(e) Whole-cell biosensors

These are biosensors based on whole living cells like bacteria, yeast, and fungi. Bioreporters have two genetic elements. A promoter gene is turned on when a target analyte enters the cell’s environment and a reporter gene is activated by the promoter to produce proteins in a process called translation. The biomarkers produced are signal-conditioned using suitable transducers for the detection of the target analytes. The use of bioreporters for detection is driven by their tolerance to environmental conditions like PH and temperature. Whole cells have more enzymes, cofactors, and substrates, and can be programmed genetically [79].

Gall et al. [80] investigated the detection of glyphosate and diuron in water using a cyanobacteria-functionalized hydrogel-gated organic field effect transistor (HGOFET). Anabaena flos-aquae, Af, strain ALCP B24 and Poly (N-alkyldiketopyrrolopyrrole dithienylthieno[3,2-b]thiophene, poly (DPP-DTT) were used as the bacteria and organic semiconductor, respectively. To immobilize alginate hydrogel on a platinum electrode, electrooxidation of EtNH2 by cyclic voltammetry was carried out. The oxygen produced by the entrapped cyanobacteria is reduced to water at the platinum surface. The resulting increased gate current is amplified at the drain. The use of photosynthesis to indicate pollutants is a low-cost method.

Silva et al. [81] fabricated an algae-based biosensor for detecting glyphosate and atrazine by peasant communities. Freshwater algae Pseudokirchneriella subcapitata and Scenedesmus acutus were used as bioindicators. Immobilized algae beads were formed by dropping alginate-cell suspension over CaCl_2_ solution. Naked eye observation of the algae growth indicated a gradual decrease in green colour, showing the sensitivity of the bioassay to the contaminants. Absorbance measurements at 465 nm yielded an LOD of 10 ppm for technical-grade glyphosate and 50 bbp for atrazine.

#### 2.3.3. Paper-Based Sensors

Paper-based sensors for pesticide detection confer many advantages, especially to developing countries whose resources are limited and which lack standardized laboratories or scarcity of experts. These sensors are low-cost, easy to operate, and can be biodegraded by microorganisms or incinerated to prevent biological hazards [45].

A paper sensor consists of hydrophilic fibres that transport the fluid through a porous medium to a desired region by capillary action. Paper is an attractive substrate because it overcomes the challenges of other microfluidic systems which demand a pump and instruments for fluid transfer. Several nanoparticle makers have been incorporated into the construction of these sensors for signal enhancement, like organic nanoparticles, colloidal gold, lanthanide, quantum dots, magnetic nanoparticles, and carbon nanotubes, among others [82]. The incorporation of nanoparticles or nanofibers into the paper matrix increases the surface area and provides active sites for pesticide binding. This enhancement promotes analyte capture and expedites the detection process. Nanoparticles facilitate rapid mass transport of analytes to the sensing sites on substrates, reducing response times—a useful quality for their integration in wireless sensor systems. Additionally, surface functionalization with specific receptors or ligands that show high affinity for the target analyte accelerates binding kinetics for faster responses. Colloidal gold is the most extensively researched, and test paper strips using this substance for the identification of pesticides are commercially available. Colourimetric analysis is the most common technique for test strip analysis where a sample is placed on a test zone and then reacts with a colour reagent, changing the colour. Interpretation of colour signals is performed by use of the naked eye, a smartphone with specific software, or a spectrophotometer measuring absorbance at a specific wavelength. Utilizing smartphone cameras or portable spectrophotometers reduces reading times, hence making the screening methods rapid, simple, and inexpensive—an admirable approach in environmental data collection. Some of the problems that need to be addressed include the environmental effect on readings, the production of readable signals, and the cross-reactivity of biomarkers [83].

Sankar et al. [84] studied the inhibition of Rhizopus niveus lipase by chlorpyrifos in their paper-based biosensor. Whatmann No1 chromatographic paper was coated with paraffin wax and then heated. Para-nitrophenol palmitate(P-NPP) and lipase were then loaded as substrate and enzyme zones, respectively. The presence of chlorpyrifos inhibited the discolouration of yellow P-NPP by the lipase. The image of the paper strip was captured by an Android phone for MATLAB processing in an open environment and a closed wooden chamber. Activity at acidic PH was lower, being 88% at PH = 5 compared to 92% at PH = 9. Up to the 15th day, the activity of the enzyme remained at 97% but dropped significantly to 87.4% after 30 days when the strip was stored at 4 °C. Metal ions and drugs inhibited the activity of the enzyme. The strip showed an LOD of 0.06 mgL^−1^.

Bordbar et al. [85] proposed a colourimetric sensor for the detection of 6 pesticides; carbaryl, paraoxon, diazinon, malathion, parathion, and chlorpyrifos. Six different NPs were synthesized for the study. AUNPs functionalized by L-arginine produced yellow and ruby-red colours, while AUNPs functionalized by quercetin produced yellow, brown, and red, and Ag functionalized by PGA produced yellow and red colours. The notable colour variations were due to the preparation methods employed. The paper pad was folded and pesticide was added to the sampling zone. After 15 s of delay, images were taken in a laboratory cabinet and analysed using Image J software. Further statistical analysis was performed for each pad. The limits of detection for each pesticide were given as follows when the assay was used on spiked samples of tap water, juice, and rice in ngml^−1^ carbaryl-29, paraoxon-22, diazinon-45, malathion-17, parathion-32, and chlorpyrifos-36.

Sun et al. [86] developed a double-film screening card for the detection of organophosphorus and carbamates pesticides. AChE and indoxyl acetate solutions were sprayed on different parts of the films. A bright blue colour would show when indoxyl acetate is hydrolysed by AChE. A digital image was taken for colour quantification and analysed using Image J software(NIH, Bethesda, MD, USA). Photoshop software (Adobe, San Jose, CA, USA) was used to extract the colour pixels. LODs in mg/mL for each pesticide were calculated at 50% inhibition concentration. Phoxim—0.05, acetate—0.5, malathion—0.5, omethoate—0.5, carbofuran—0.04, and aldicarb—0.08. The efficiency of the card was 75% when stored at 4 °C, and this decreased to 50% at 37 °C.

**Table 1:** Advantages and disadvantages of the techniques used for pesticide detection in water.

Method	Merits	Demerits	References
High-power liquid chromatography (HPLC)	Can analyse small organic molecules, large polymers, and biomolecules. Its combination with mass spectrometry (MS) gives it the perfect ability of separation, increasing sensitivity to trace amounts and specificity. Excellent separation efficiency Detection results can be reproduced.	Not easy to teach to a new person Large quantities of expensive organic compounds are required Laborious calibrations and sample preparation especially for routine analysis.	[87]
Liquid chromatography-mass spectrometry (LCMS)	Good linearity High recoveries and precision Low LOD and LOQ Can analyse many pesticides compared to GCMS Can exclusively detect carbamates and triazines. Can detect OC better	Requires expensive equipment Requires a specially trained technician	[88,89]
Gas chromatography-mass spectrometry (GCMS)	Highly sensitive to non-polar, volatile pesticides Can be used selectively or universally Has two modes of operation (full scan and selected ion-monitoring (SIM)) Good response	Can analyse fewer compounds compared to LCMS Sample preparation needs derivation Injection head temperatures decompose some pesticides Responds to impurities in the carrier gas, air leakage in the GC system, or stationary bleeding from the column	[90]
UV-VIS-NIR	Allow rapid measurements Fast response Chemical free Non-destructive Low operating costs Environmentally friendly Reduced reagents required for analysis	Preconcentration steps are required Near infra-red (NIR) spectra are more complicated to analyse due to the combination and overlapping of vibrational modes Multivariate techniques required for analysis Lower sensitivity Light scattering by suspended solid particles causes serious errors Matrix interference Longer duration of tests UV is harmful to humans	[46,50,91,92,93,94]
RAMAN	Water and glass show low Raman scattering Non-destructive Does not need complex sample preparation Symmetrical bonds show strong Raman while they are inactive in infra-red Shows better spatial resolution than IR A single instrument is used in the entire vibrational mode while IR uses different Useful in the analysis of unknown substances	Fluorescence of impurities is very strong. Raman spectra are weak Expensive instruments	[95,96]
SERS	Simple pre-treatment Selective in detecting complex environmental pollutants Signal amplification Quick analyte identification using fingerprinting SERs spectra Portable appliances	SERS substrate deteriorates over time. High cost Affected by matrix effects Poor reproducibility	[97,98]
BIOSENSORS	High sensitivity Fast response Robust Low cost Miniaturization In situ and real-time monitoring High specificity Applicable in complex mixtures Simple operation	Qualitative or semi-quantitative results Legal limitations for genetic modification of organisms Some biological materials can be denatured by the environment (PH, temp, ions) Most devices are still in the laboratory stage	[56,61]
Paper-based sensors	Non-expert operation Portable Easily disposable Low detection limits Fast response High reaction speed High specificity Multiple analyte identification Small volumes of reagents	Short shelf life of a few days Some have poor stability Most devices still in laboratory stage Long fabrication time	[99,100,101,102]

## 3. Internet of Things for Environmental Monitoring

### 3.1. Internet of Things

**Figure 2:**
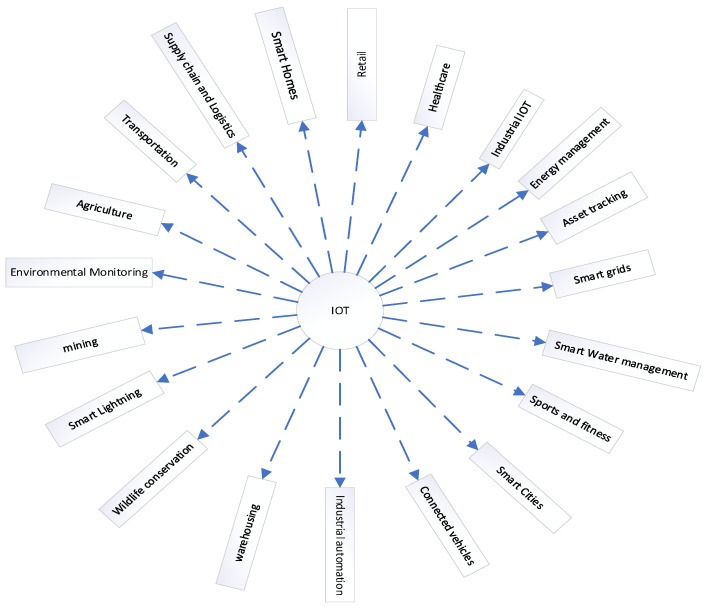
The IoT Hub Nexus map.

Nowadays, the Internet of Things is shaping the landscape of operations in many sectors by interconnecting systems. Industries can now optimize their processes by utilizing and expanding their remote operations [103]. A variety of domains have seen the deployment of IoT products, as depicted in the Figure 2 below: smart grids, vehicle-to-everything, wildlife management, supply chain logistics, and smart healthcare, among other cases [104,105,106].

Sensors are the primary components of IoT systems designed for Machine-to-Machine (M2M) communication, serving the purposes of monitoring or actuation. Temporal and spatial information concerning environmental conditions can be easily gathered and transmitted for analysis by organizing these sensors in networks [26,107]. However, there is a limited exploration of IoT connectivity for pesticide sensors in the domain of water testing. This is mainly attributed to the unavailability of plug-and-play sensors that can be directly connected to other platforms. Published work shows authors interface their developed prototypes to the internet for management purposes. Using a multispectral sensor AS7265x, Ajira et al. [108] analysed the presence of glyphosate in spiked lab water in their device, SpectroGLY. The presence of glyphosate is based on colourimetric chemical reactions detectable using reflectance spectroscopy. Its response was in the form of traffic lights with unspiked samples producing green light, while water containing 10–499 mg/L of glyphosate triggered yellow light, and red light indicated levels of more than 500 mg/L. A mobile application connects to the device via Bluetooth low energy (BLE) and allows users to enter parameters, including the water source and reagents used. The control in the mobile phone allows users to request tests, as well as change communication configurations. Wi-Fi is chosen in urban areas, and LoRAWAN is preserved for rural setups. Additionally, MQTT and HTPP protocols are used for communication with The Things Network (TTN), enabled by MCU ESP32 and Heltec Wi-Fi lora modules. Smartphones give users GPS coordinates in the dashboard display, along with additional information like sample results and their spectral signatures.

The use of smartphone resources as a signal collector, processor, and displayer in pesticide sensing is common in studies [109,110,111,112,113,114,115,116]. User-designed applications are used to detect colourimetric, fluorescent, and chemiluminescent signals for analysis, while others detect voltage and current variations [117]. Some of these developed applications upload the same data to the internet for public viewing. Sicard et al. [118] designed a paper-based analytical device for the detection of malathion in water. The paper sensor was based on the inhibition of acetylcholinesterase enzyme by the pesticides. Two images (a test strip and a control) were captured using an iPhone installed with an algorithm for colour determination. The images were geo-tagged for identification of the site where the test was performed. The results were accessible via a website named WaterMap Ca. The application allowed the uploading of location coordinates and measurements. The HTTP protocol was utilized for uploading the data to be stored in the MySQL 5 database. Before submission, users register an account on the WaterMap website, and a random token is supplied to them for any data point to be uploaded. Data points are displayed with the date, name, pesticide concentration, and GPS coordinates of the site of the test.

Vaseashta et al. [119] developed a network of connected sensors to monitor several environmental contaminants, including metals, pathogens, organic compounds, pharmaceuticals, and pesticides. Commercial off-the-shelf sensors capture data and communicate to a control centre for synthesis and analysis. GIS sensors were used to provide the location of sensors or where samples are taken. Monitoring and identification of chemical contaminants in the groundwater of the Prut River basin was performed using gas chromatography atomic absorption spectrophotometry, electrochemistry, and laboratory water analysis with conductivity sensor SE6780/G1. To demonstrate compliance with chemical status, EU and US EPA standards were applied.

As we edge closer to full IoT integration in pesticide monitoring of water ecosystems, existing studies in other fields are already pointing to issues in the list of redress. There are efforts to optimize battery life to enable the nodes to run for years with minimal charging or to incorporate energy-scavenging modules while designing the sensors [120]. This impacts the reliability of data due to the increased uptime of the nodes. With more devices being connected and exchanging information, security compromise is a threat that needs to be addressed. IoT devices are becoming vulnerable to different types of attacks like spoofing, Botnets, Denial of service (DOS), or Man-in-the-middle attacks [121]. Appropriate measures should therefore be taken to safeguard these networks from cyber-crime by implementing zero-trust security models, data encryption, or the use of digital signatures [122]. Enhancing security will build confidence in users, especially in situations when personal data is to be shared within platforms.

### 3.2. Communication Protocols

#### 3.2.1. Message Queuing Telemetry Transport (MQTT)

MQTT is a lightweight messaging protocol designed for constrained devices [123]. These are low-bandwidth, high-latency, limited-memory devices, or devices operating in poor networks [124]. The protocol works in the publish–subscribe model and depends on brokers. A client publishes a message on a specific topic with the broker, and then the device(s) that have subscribed to the topic will receive the message. Its messages have three Quality of service (QoS) levels: (1) “at most once”/“fire and forget”; (2) “at least once”/“Acknowledge delivery”; and (3) “exactly once”/“assured delivery” [125,126]. The protocol relies on the Secure sockets layer (SSL/TSL) for security, since it is designed for secure networks and runs on Transmission Control Protocol/Internet Protocol (TCP/IP) in the transport layer.

#### 3.2.2. Hypertext Transfer Protocol (HTTP)

This is a stateless protocol where clients use request-response commands for data communication over the World Wide Web [124]. The requests have big headers containing more information than the body of the message, which ends up straining the network [127]. The pooling nature of this model and lack of optimization for IoT devices leads to high power consumption. Its secure version, HTTPS, is encrypted by SSL/TLS for data integrity and confidentiality. It uses GET, POST, PUT, and DELETE to perform operations on resources [128].

#### 3.2.3. Constrained Application Protocol (CoAP)

The CoAP is a client-server model designed for nodes with low memory, limited processing power, or in lossy networks. The CoAP supports four types of messages (Confirmable (CON), Nonconformable (NON), Acknowledgement (ACK), and Reset (RST)), which are exchanged asynchronously in request-response commands [123].

Security is ensured via Datagram Transport Layer Security (DTLS), utilizing User Datagram Protocol (UDP) as its underlying transport protocol [129]. This selection minimizes overhead but introduces a degree of message unreliability, characterized by potential packet loss, unordered arrivals, or duplication.

#### 3.2.4. Advanced Message Queuing Protocol (AMQP)

This is a binary protocol designed for interoperability and supports both publisher/subscriber and request/response architectures [126]. AMQP is a message-oriented middleware (MOM) with three QoS (At most once, at least once, and exactly once) and ensures reliability by storing messages in queues in case of network disruptions [129]. The protocol uses Transmission Control Protocol (TCP) for communication and is secured by SSL/TLS or Simple authentication and security layer (SASL).

#### 3.2.5. WebSocket

This is a full-duplex communication designed to provide real-time communication between the server and clients [123,130]. The client initializes a conversation through an HTTP request for a handshake, and, once connected, messages are delivered asynchronously over TCP. The messages have reduced headers, minimizing bandwidth usage, and can be forwarded sequentially in a data stream. Regular connections use port 80, while TLS-secured connections use port 443, just like HTTPS.

#### 3.2.6. Extensible Messaging and Presence Protocol (XMPP)

This is an instant messaging protocol mostly used for chat, voice, and video calls [124]. The exchange of data is performed in Extensible mark-up language “(XML) stanzas”, a piece of code containing a message, presence, and info/query (IQ) [128]. XML stanzas are known to add processing overhead.

### 3.3. The Cloud and Computing Models

The cloud is an essential component in the IoT ecosystem, as it provides additional space for the storage of data and applications [131]. IoT devices are constrained in terms of memory and processing power. The data gathered by IoT devices can be comfortably accommodated in the cloud, as having more space saves the IoT device the burden of putting up with too much data. Complex applications that require high processing speed can be stored in the cloud, and can only be called when required by the devices. This will allow them to run optimally and be efficient in data collection [132].

#### Computing Models

The need for data warehouses/data lakes, the high cost of maintaining hardware for medium enterprises, and the demand for a great deal of services or applications led to the invention of computing models. Several computing models are currently at our disposal, like cloud computing, edge, fog, and mist computing.

(a) Cloud computing

Cloud computing involves pushing vast amounts of data to remote servers for analytics and storage [133]. It is an invaluable tool in innovations that generate large data for processing as the cloud provides the necessary infrastructure and is scalable, enabling millions of devices to connect and share resources. Some studies have, however, noted security concerns for uploading data to the cloud when many devices have access [132]. The handling of massive amounts of data introduces a whole set of challenges, like load balancing, low latency, high network bandwidth, poor real-time analytics, poor reliability, and high energy consumption [134,135].

(b) Edge computing

The shortcomings of cloud computing led to the development of edge computing by taking computations to the proximity of data sources [136]. This paradigm is mostly suitable in real-time analysis where quick decisions are required, like in vehicle-to-everything and Unmanned aerial vehicles (UAVs) [137]. The evolution of the 5G network allows edge computing to bring cloud services closer to the users.

(c) Fog computing

Fog nodes are distributed over large geographical areas with onboard storage facilities and computational resources [138]. The existence of these resources determines what can be done locally and what needs to be sent to the distant cloud. One of the unique features of fog is the possibility of data availability in offline status, which adds to security and privacy concerns as the data components bearing fingerprint identities of users can be analysed and extracted before onward transmission to the cloud [139].

(d) Mist computing

Mist computing lies at the extreme edge of the network [140]. It includes microcontroller capabilities of collecting raw data, recognizing patterns, or performing filtering and data cleaning before onward transmission towards the cloud [139]. The forwarding of only essential data to routers or gateways saves the energy required in the communication process.

## 4. Wireless Sensor Network for Water Quality Sensing

**Figure 3:**
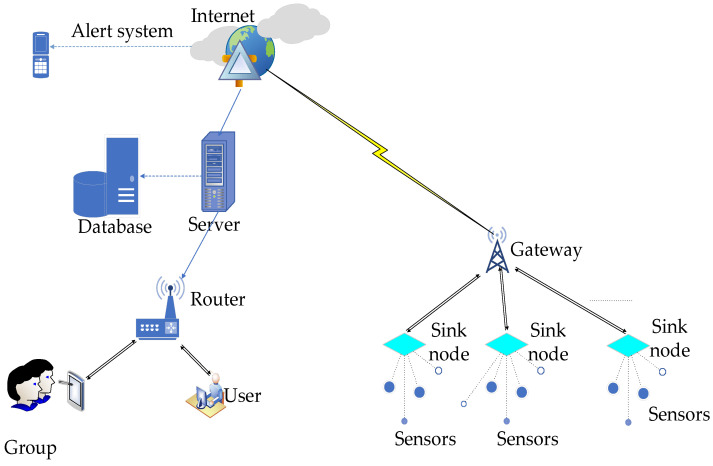
A wireless sensor system.

A wireless sensor network is a collection of tiny, low-cost electronic devices capable of gathering data and forwarding it to a base station [141]. Figure 3 shows a typical layout commonly used in monitoring scenarios. A major constraint that is observed in the design of a network is energy demand; since these sensors use low-power batteries for communication, energy-efficient mechanisms need to be employed [142]. However, despite this challenge, WSNs have become a revolutionary technology in monitoring due to their increased intelligence. Additionally, wireless sensors have several advantages, like scalability and flexibility, and can be centrally accessed and do not need wires or cables [143]. These strengths have propelled them to be true assets in the fields of agriculture, health, smart homes, military, and space exploration. Due to the existence of various wireless technologies, a careful selection is needed to obtain optimum benefits from the chosen network. Studies have shown that the most commonly used technologies in monitoring aquatic environments are Radio Frequency Identification (RFiD), Bluetooth, Zigbee, radio 802.15.4, Wi-Fi, Long Range (LoRa), Narrow Band Internet of Things (NB-IoT), General Packets Radio Service (GPRS), and Long-Term Evolution (LTE) [144]. RFiD is a dedicated short-range communication method that does not need a line of sight to uniquely identify items. RFiD tags can store substantial amounts of data and allow simultaneous readouts. When several tags are read at the same time, data collision can occur, leading to a loss [145]. Bluetooth is another short-range communication protocol commonly suitable for low-powered devices. This protocol operates in 2.4 GHz frequency of the unlicensed industrial scientific medical (ISM) band, using frequency hopping modulation supporting point-to-point and point-to-multipoint voice and data. Water molecules are absorbed in the same frequency band, and microwaves also operate in that frequency, thus limiting Bluetooth usage in water and possibly leading to interference from microwaves [146]. ZigBee devices can communicate over distances ranging from 10 to 100 m. This distance can be increased significantly using mesh networks, a common topology employed for these devices, though power consumption is higher due to the additional routers [147]. A Wi-Fi access point spans an area of up to 1 km radius. Wi-Fi networks are occasionally flooded with large amounts of data, and unintended users can gain access or cause interference due to poor encryption [148]. Cellular or mobile networks are characterized by high cost and high data throughput, reaching speeds of several Gbps, and low latency. They consist of various standards like General Service Mobile (GSM), LTE, General Packets Radio Service (GPRS), Code Division Multiple Access (CDMA), Enhanced Data for Global Evolution (EDGE), and 5G [149]. The devices operate in Frequency Division Multiple Access (FDMA) or Orthogonal Frequency Division Multiplex (OFDM) and need frequent synchronization, leading to high power demands. However, one of the variants known as NB-IoT has low data rates and uses extremely low power, rendering itself useful in monitoring applications. LoRa is a communication technology that utilizes global free bands 433, 868, and 915 MHz. This protocol uses a chirp spread spectrum, spreading the bandwidth and making it resistant to multipath and fading [150]. Additionally, its devices are asynchronous and use Advocates of Linux Open-source Hawaii Association (ALOHA) in communication, thus increasing the battery lifetime [151]. LoRa devices have a range of up to 15 kms and use two keys for authentication; however, their data transmission rate is low at 50 Kbps [152,153]. Several groups have carried out research in aquatic environments using these technologies to investigate various parameters [154,155,156,157].

Jen-Yung et al. designed a system for monitoring several water parameters using sensors integrated into the ESP-WROOM-32 Wi-Fi module [158]. The monitoring data were relayed to the Thing Speak IoT and viewed via the Thing View APP. Message queuing Telemetry Transport (MQTT) was used as a messaging protocol to the ThingSpeak cloud and Hypertext transfer protocol for the application layer. Data streams were visualized and analysed using MATLAB. Software design was performed under an open source Arduino-integrated development environment supporting both C and C++.

Huan et al. monitored water quality for aquaculture ponds using NB-IoT [159]. The data were sent serially by the MCU to the NB module, encapsulating the payload into the message of the constrained application protocol, and then forwarded to a preconfigured IoT Telkom cloud platform. An STM32L151C8 chip with an ultra-low power Arm Cortex-M4 CPU was connected to four sensors. Its internal timer controlled the sending of data every 30 min, and the internal ADC converted the analogue values of PH and dissolved oxygen to digital. The communication module was Yiyuan BC95-B5, based on the NB-IoT which uses the 3GPPRel.13 protocol operating in 850 MHz. To facilitate the parsing of data, IP addresses and International mobile subscriber Identity (IMIS) were added to the data packets. When the platform received a command, it was converted to a HEX decimal code stream and sent to the end device for intelligent control, while reported data were stored for monitoring. The packet loss rate from cloud data was 0.42% for a communication range of 3 km between the node and the gateway.

L. Vacariu et al. designed a real-time water parameter monitoring system using a LabVIEW hierarchy [160]. Measuring nodes sampled the quantities from the sensors and conditioned them before transmitting them to the gateway using the 802.15.4 radio protocol. The gateway communicated with the measuring nodes using a short-range protocol of less than 1.6 km. The data received by the gateway was stored using the FIFO structure and was available on a web server accessible via the HTTP protocol. Data transmission to the gateway consumed a great deal of energy, and thus it stayed disabled until the time to transmit.

A. M. C. Ilie et al. [161] built a WSN using three Waspmotes (sensor nodes) and a gateway called Meshlium for monitoring pH, temperature, ORP, and EC. Waspmotes are low-power devices immune to water and dust, but which lack a storage facility. They were equipped with a 6600 mAh battery and a solar panel of 2 W. This made them autonomous and easily deployable. The Waspmotes package also included an Atmega 1281 processor with 32 KB RAM and a 14 Mhz clock. A low-power 802.15.4 radio covering a few hundred meters (line of sight) sent the data to Meshlium. The Meshlium acted both as a data logger and gateway featuring several interfaces, including an 802.15.4 radio, RJ45 ethernet, Wi-Fi, and a GPRS/3G modem. It had an x86 CPU running Linux OS with 256 MB memory and 500 Mhz clock speed. The data sent by the Waspmotes was stored in the Meshliums database using the Frame Parser, and it was possible to synchronize it to an external cloud partner.

Faustine and Mvuma [162] worked on a Bluetooth wireless system to detect water parameters in Lake Victoria. The sensors were connected to an ATmega 256 microcontroller with 70 I/O and powered by a 3.7 V 6AH polymer lithium battery. To enable communication with the U-WQR Mobile Application, the Android Open Accessory Protocol was applied. A connection was established via the serial Bluetooth, which works with USB. The system provided mobile reporting of water quality in graphical form to stakeholders. It was noted that some parts of rural areas lacked sufficient network coverage, which limited instant data deliveries.

Singh, S. et al. [163] deployed an IoT RTWQMS (real-time water quality monitoring system) in the river Ganga in India. The system monitored 17 quality parameters and consisted of a sensor package, a data processing and publishing server, and a GPRS for communication. The system was based on SCADA (supervisory control and data acquisition). A data logger connected to a router received the data gathered by the sensors and sent it to a central database in real-time. Data validation was performed with laboratory values obtained at the time of deployment.

Geetha designed an IoT system to monitor turbidity, water level, and PH [164]. A single-chip microcontroller TI CC3200 with an in-built Wi-Fi module and ARM Cortex M4 core was used. Sensors were directly interfaced with the controller and the data gathered was uploaded to the “Ubidots” cloud. The platform had a dashboard that allowed data analysis and sent SMS alerts to concerned persons for action when threshold values were exceeded.

K. A. U. Menon et al. [165] proposed a wireless system for monitoring PH in a river. A hierarchical topology was adopted for the monitoring. The sensor nodes transmitted gathered data using Zigbee to cluster heads. Zigbee was notably cheap and had low power consumption. Outdoors, the range of transmission was approximately 70 m. A Pic microcontroller PIC16F877 was used for the aggregation of data from the sensor nodes. This microcontroller had an inbuilt ADC, reducing the cost and power needs. A UART serial interface sent the data to the communication module, a chipcon CC2420 radio transceiver operating on IEEE802.15.4 standard. The data rate was 250 Kbs, operating in the 2.4 GHZ ISM band. The data were then wirelessly transmitted using Zigbee to the Mote at the base station for analysis.

D. Nguyen and P. H. Phung [166] presented the design of a WSN to monitor environmental conditions with minimal data packet losses in transmission. Sensors were powered with 2AA batteries. The smart evaluation board RF05 was the coordinator and was connected to the SIM 300CZ GMS/GPRS module via RS 232 as a UART. After setting up the Zigbee network, it would receive data from the node and check its ACK number. New data would be forwarded to a server through a TCP/IP link. Additionally, an SMS would be sent to users for any data falling outside the appropriate range. Connection failure would cause the data to be stored in an NV flash memory device (NV CC2530) to await reconnection. Users could query the server using the client user interface, built on the Windows OS.

Jia [167] developed a WSN with two types of multisensory combination modules (MSCM): one for water, and another for air. Each MSCM had a GPS sensor attached to identify the problem location. The system used an FX1278 Lora remote modem with a power consumption of 100 mW and a sensitivity of −148 dBm. The module operated at 433 MHz and was connected to seven sensors. Data acquisition nodes were connected to the STM 32 processor through I/O pins. The processor was noted to have a low power consumption. Another similar processor was used for the base station which uploaded the data to the cloud via LTE. A queue-based data upload algorithm was employed utilizing the TDMX technique. To save energy and prolong the lifetime of the network, data fusion based on fuzzy decision-making was carried out at the collection node.

Sendra et al. [168] fabricated a LoRa-based network for monitoring coastal water quality parameters, including water temperature, turbidity, and humidity. A data storage integration service by The Things Network was included. This data in the database could be extracted and visualized using Ubidots. The gateway operated between 868 MHz and 915 MHz, and its antennae had 14 dB gain. The Things Uno with LoRAWAN RN2483 Microchip was used, which is compatible with Arduino Uno and existing shields. The protocol for accessing data in the server was HTTP, both of which are supported by TTN. Using the received signal strength Indicator (RSSI), the maximum distance that a node can send data was 910 m while working at 968 MHz.

Qian et al. [169] presented the design of a flexible RFID tag for sensing minerals in water using a smartphone. The front-end sensing module was a capacitive-to-digital converter CDC chip PCAP02 with a power supply of 2.1 V and 2.5 µA. A radio frequency harvesting module HF RFID chip M24LR16E was used for efficient inductive coupling, with low power demands capable of producing 2.1 V/1 mA max power output. The chosen MCU MSP430FR2433 operated at 1.8 V 126 µA/MHz and 1 µA/MHZ in active and standby modes, respectively. When an interrogating smartphone equipped with an HF RFID taps the sensor tag, sensor data is instantly displayed on the screen as an NFC message. Sensor data transmission was performed efficiently through the NFC data exchange format. Carrying out TDS tests using the proposed method revealed that the sensitivity of the capacitor decreased with increasing mineral concentration.

The work presented in [170] proposes the design of a WSN based on GPRS. A total of four water quality indexes were monitored using composite electrode sensors. A sensor node was constructed using the STM32F103 microcontroller programmed in C language. A low-power FSK-based RF transceiver A7139 was chosen for communication. This module offered a 1000 m range and 100 mW power consumption during transmission. The gateway node had a similar processor, along with a serial communication module and an SD card for storage. The flooding routing protocol was chosen for its effectiveness and robustness; however, a registration sheet was created in the memory of every processor node to prevent network congestion challenges associated with flooding. The packet loss rate was evaluated to determine the network reliability and found to be 3.55%.

The authors of [171] evaluated the performance of the LoRa-based system in the detection of water pollution based on its PH and temperature. In their assembly, an Arduino Uno board was used alongside a Dragino Lora shield for the transmission of detected values to a cloud. Three compounds (Phosphate, Potassium Hydrogen Phthalate, and Sodium tetraborate) were used for the measurement of the different PH values. RSSI values were determined for different distances measured using Google Maps. For 3 km, an RSSI value of −29 dB was obtained. For 7 km and 10 km, the RSSI values were −77 dB and −116 dB, respectively. The PH values measured were validated with the voltage values given in the data sheets.

Parra et al. designed a water quality monitoring system for coastal areas [172]. They designed the sensor node using Arduino Leonardo and three sensors, namely salinity, turbidity, and temperature sensors. They designed a salinity sensor using two solenoids, an enamel copper wire of 0.4 mm, and a PVC tube with a diameter of 2.6 cm. A 7555Ic was used to generate a square wave for powering the sensor. For turbidity measurement, an RGB LEDS arrangement was established alongside an LDR. The three LEDs were powered from the digital pins of Arduino. An RTC DS3231 was used to add a time tag to gathered values and also set the node to make measurements on an hourly basis. This timer is not affected by variations in temperature. An ESP 32 module was used for communication. The sensors were calibrated using volumes of 100 mL. Measurement was performed by introducing the samples into the pipe and carefully extracting them using pipettes. The node was kept in the dark to reduce interference from ambient light. Multivariate techniques were used for analysis of the results.

A. Belsare et al. [173] monitored the quality of water using a floating module equipped with various sensors. The system gathered the PH, temperature, and turbidity of the water. An ultrasonic sensor was also included to monitor the distance of the floating device from the water surface. A WIR-1186 module was used for communication. Its range was a 2 km line of sight. A PIC18F2520 microcontroller was used for data processing. At the receiving end, a single push button on the GUI made the system acquire the environmental data and store it in an Excel format.

The work presented in [174] proposes the design of an array of electrochemical sensors for monitoring pH, free chlorine, BPA, and temperature in water. The read-out circuit consists of Arduino Uno with stacked water quality monitoring and potentiostat boards. These two boards contained the circuits associated with analogue and mixed-signal sensors. A Bluetooth transceiver was connected to the board for communicating wirelessly to a custom Android application in a smartphone. BPA sensing relied on Voltametric measurements. The smartphone application consisted of an interface to upload the measured data to an online storage. The sensitivity of the PH potential metric sensor was very high (57.5 mV/pH) while the chlorine was 186 nA/ppm. An Android application named Smartphone Suite was developed in Java, and on opening would prompt the user to select the kind of measurement to run. The screen also displayed three graphs corresponding to the sensor outputs. Sensor data were updated every second and historical data accumulated in time graphs.

Lastly, Razman et al. [175] reported the design of a water quality and filtration system. Sensors were used to detect the PH, oxidation-reduction potential, turbidity, temperature, and electrical conductivity. Arduino Uno and Mega formed the processing unit, while the data transmission block was the ESP8266 Wi-Fi module. A water pump and filter made up the filtration unit. After processing, the data were uploaded to the cloud and could be monitored on the ThingSpeak platform. If the values from sensors indicated that the water was not fit, then it would be sent to the filtration block. Box plot analysis is used to identify abnormal data. One-way ANOVA is also applied in the analysis. A box plot of three water samples was performed for lake, tap, and river water.

## 5. Innovations in Pesticide Detection and Environmental Analysis

### 5.1. Machine Learning for Enhanced Pesticide Detection

The advances made in artificial intelligence are growing daily due to the introduction of machine learning as a new tool in that domain. As an advanced form of statistical analysis, ML involves predictions and decisions based on data. Accurate predictions depend on the information fed into the system. There are various types of supervised and unsupervised machine learning methods. Supervised methods require more human input when training on data using superimposed labels [176]. This involves the development of predictive models based on patterns and relationships between inputs and outputs. Some common algorithms in this category are Logistic Regressions, K-Nearest Neighbours (KNN), Random Forests (RF), Support Vector Machines (SVMs), and convolutional neural networks (CNNs) [177]. Unsupervised machine learning can figure out things that have not explicitly been stated [176]. The models in this category are Principal Component Analysis (PCA), Gaussian mixture models, Apriori algorithm, and Hierarchical temporal memory [177]. Other techniques, called Ensemble methods, have been developed that combine predictions of multiple models to improve the performance and give better predictions. Bootstrap Aggregating, Adaptive Boosting and Voting Classifiers fall under this category [178]. Many sectors of the industry have been able to benefit enormously from this tremendous leap in technology. The employment of machine learning algorithms for the identification of parameters in complex environmental situations has yielded interesting results, as suggested by [179] by using the binary classification algorithm SVM to maintain the quality of data for environmental sensors deployed in very noisy environments instead of performing routine preventive or corrective maintenance for obstructed sensors.

In the development of the models, the focus should be on the quality of training data, feature selection, and identification of appropriate algorithms. The authors of [180] used ML to identify four pesticides on Cedrus Libani’s paper using silver nanoparticles. The Raman spectra are used in their original form without pre-treatments. A total of 583 samples are collected for the experiments with the following distribution: myclobutanil 122 samples, thiram 167, abamectin 151, and phosmet 143. After the reduction in dimensionality using PCA, five models were used for classification: SVMs, KNNs, Decision Trees (DTs), and AdaBoost, all giving prediction accuracies of more than 88%. KNN is considered a traditional method and had the highest success rate of 93%. The authors, however, noted that classification accuracy could be improved by creating a database with more pesticide types. A similar observation regarding the use of larger samples for more robust models was made by [181] while comparing the performance of conventional ML methods (LR, SVM, and RF) with deep learning CNN and ResNet methods. In a study to detect pesticide residue on grapes using hyperspectral imaging, the deep learning method ResNet produced better results than the other models for both VIS-NIR and NIR spectra. Overall, all the models had accuracies of over 90% in training, validation, and prediction, except for RF which showed overfitting in both spectra ranges.

In ref. [182], the authors designed an optimized Resnet-based deep learning model to classify multiple organophosphorus pesticides in environmental water. Other co-existing compounds are known to mask the SERs signals, hence their identification through visual inspection is limited. A total of 9000 multicomponent samples of the SERs spectra were collected for the training model using an established multicapture SERs platform and split at 90% and 10% for training and testing, respectively. The model achieved close to 100% accuracy for the classification and regression, while results from the confusion matrix posted greater than 97% accuracy in classifying all six Organophosphorus pesticides.

### 5.2. Big Data Analytics in Environmental Monitoring

The monitoring of environmental parameters calls for the deployment of many sensors spanning the area of interest. Thus, IoT sensors for data collection generate large data sets, often coming from a variety of sources in real-time. As such, IoT data is referred to as big data having variety, volume, and velocity [183]. With some suitable integration mechanisms, these datasets can be used to provide more insights and hence deliver more useful information. Analytics is the conversion of raw data by the use of tools and technologies into actionable insights by finding trends and patterns [184]. To facilitate aggregation, cleansing, storage, and analysis of massive data, technologies such as Spark, Apache Hadoop distributed file system (HDFS), MapReduce, NoSQL databases (MongoDB, Redis, and Hbase), Hive, Kafka, and Storm have been suggested [185]. In the field of environmental monitoring real-time sensors, IoT and social media platforms produce a large variety of data whose analysis provides crucial information on the existing conditions. Using knowledge discovery in the database, ref. [186] monitored 170 plant protection products (PPPs) to identify residues in water surfaces. The study led to the identification of nine main PPPs in the Puglia River, with Glyphosate, AMPA, Imidacloprid, and azoxystrobin as the dominant residues. Samples for quantification were analysed by UPHLC-MS/MS with a 3-month sampling regime stretching for 4 years. A multidisciplinary approach was applied for analysis, including heatmaps, cluster analysis, GIS analysis for land use, and Apriori algorithm for data mining. A total of 1461 PPS from 2018 to 2021 were reported, with Glyphosate and AMPA being the most common in three years (2021—62%/38%, 2020—21%/36%, and 2019—18%/36%). The data mining results allowed the identification of mixtures containing up to nine compounds, while correlational heat maps on normalized scales presented easily readable patterns. In-depth discoveries like the presence of Glyphosate and AMPA in lagoons in the first quarter of the year were explained by rainfall data from weather stations. Big data is adequate for training ML models to have high accuracy in prediction; however, cleaning is a vital step in the pre-processing stage, as data is unstructured or in a semi-structured format. The complexity of these formats requires good curation and storage, as well as advanced statistical approaches to extract relevant information. While monitoring 165 water fields with approximately 25,000 samples at a time, ref. [187] included a module for data cleaning in his water quality monitoring system. The module was designed to determine the validity of data before storage by removing outliers. The group noted that captured data may be non-numeric, wrong, or missing. The missing values were obtained by performing linear regression fitting interpolation. After the clean-up process, the values were stored in the MySql database, a relational database management system used for web applications. Analysis of the data was performed using influxDB due to its open-source nature and suitability for time series data. To cut costs, they rented Tencent Cloud for computing resources and visualized the data using Baidu Echarts and WeChat for communication with users. The dimensionality curse is a common phenomenon while dealing with big data which needs to be dealt with during data processing [188]. According to Zhong et al. [189], big data slows down the processing speed of data. Therefore, they explored improving on the existing Fuzzy c- means clustering algorithm while analysing water quality data in the Three Gorges Reservoir Area. The algorithm was meant to put a distribution of data points in multidimensional data space into several classes. The suggested fast Fuzzy C-means clustering algorithm reduced the number of iterations in classifying 1024 sample data from 44 steps to 24, speeding up convergence and improving efficiency.

## 6. Integration of Technologies for Comprehensive Solutions

**Figure 4:**
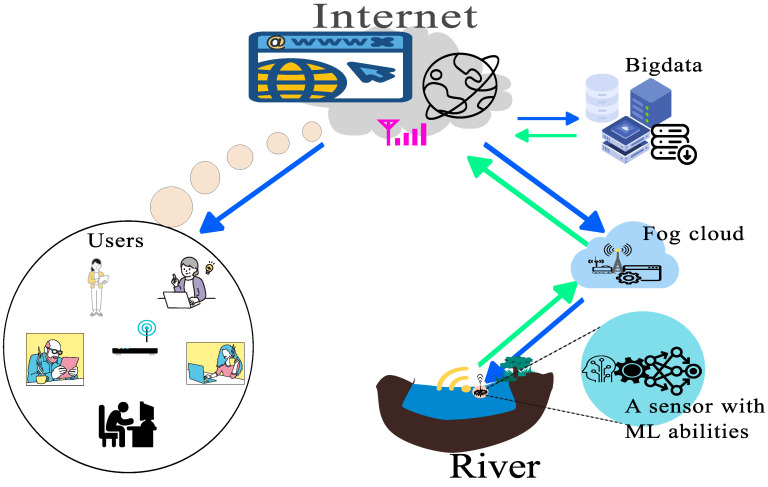
Integration of technologies in pesticide sensing.

Several benefits can be realized when WSN, IoT, ML, and big data are integrated into monitoring pesticides in water. A high degree of granularity of information concerning the water ecosystem is obtained. Aggregation of data concerning the weather patterns, topography, and the history of contamination levels alongside pesticide determination will offer a more holistic view of the contributions of the other players in the water ecosystem. Each of these methods used in the measurement of pesticides has its inherent advantages and their combination will reinforce their capabilities, further stretching their horizons beyond criticism. Figure 4 illustrates an envisaged integration that holds promise for enhanced pesticide detection outcomes.

WSNs have for a long time been lauded for providing more spatially distributed data due to their inherent property of being deployed over large geographical regions and difficult-to-reach areas. When coupled with IoT infrastructure, the collected data extends beyond mere snapshots of large environments. The connectivity and diverse capabilities offered by various applications within the cloud enable the realization of multi-modal data integration. With more studies following the route of integration, well-detailed and synthesized results will become available. Even though the integrations have happened in the detection of other water parameters, their application in pesticide sensing can bring the required and necessary change in the domain of pesticide monitoring.

The authors of [17] apply several approaches while monitoring pollution along a river basin water. Using Libellium Waspmotes, they monitored pH, temperature, Oxidation-reduction potential, DO, conductivity, fluoride ions, nitrate ions, and calcium ions. The sensor nodes were connected in a star topology to a base station to minimize latency while the GSM modem uploaded measured data to the cloud from locations where stakeholders could view the analysed data. The sensor node architecture was partitioned into a sensing sub-system, power, communication, and computing, which also had a prediction of power requirement. Sensor nodes were programmed to send data once every 30 min, while cloud uploads would be performed once the connection was established; otherwise, they would revert to sleep mode to save energy. A system to indicate received packets by base station was established, although sometimes a packet would be sent more than once due to failure of acknowledgement. One of the challenges they faced was biofouling; they realized the common methods of removing, like chlorine evolution, and use of wipers or copper, were not applicable in their case. Other challenges included damage to sensor probes, untimely recharge of data packets from service providers, and malfunctioning equipment.

Edge computing is instrumental in WSN monitoring, especially in situations where large areas are involved. Park et al. [190] noted in their study performed in the river Keum for green tide prediction that while ML/DL has been used in many studies involving centralized cloud computing to predict pollution in rivers, the models do not accurately fit in certain cases. The authors proposed federated learning (FL) because the sensors were being deployed over a very large geographic area. If the sensors send data to a central cloud, measurements will sometimes arrive unsynchronized, due to network degradation and the distances involved. The study proposed several edge servers along the river which would receive data from the sensors and then use it to train a local model. They then forward their optimized local parameters to a central cloud in synchronized unit time for aggregation and computation of a global model which will be passed to the edge servers. Barrages provide power to the local servers along the river and smart sensors are deployed close to them, sending their packets using NB-IoT. The algorithm for FL in the distributed network avoids imbalance in local training data and allows some edge servers to continue local learning, with data quantity unsatisfactory for target performance. He proposed an edge queue model with a feature to store all the data received by an edge server in a queue and use it for learning, even if it exceeds the threshold data for a single iteration training. In situations where the data received is less than the baseline, the server waits for the next scheduling to meet that requirement. Seven real-time water quality indicators, including Dissolved Oxygen (DO), Total Organic Carbon (TOC), Total Nitrogen (TN), Chlorophyll-a (chl-a), PH, turbidity, and temperature were employed to train the model. In the interest of simulation, data spanning a five-year period retrieved from state-managed sites was utilized. The authors noted that setting the number of values of collected data which can initiate training prevents overfitting and underfitting. The accuracy of the model increases as the number of values required to initiate learning increases from 20. It rises sharply to 80% for 50 values, then converges to greater than 90% for data values beyond 100. However, increasing this reduced the number of edges that performed the training, thereby negatively impacting the performance of the model even though good accuracies were attained. High-performance models can be obtained with a large baseline. However, this can only happen if the baseline exceeds the threshold sample data, in which case it is impossible for many edge servers to train. It is clear from this study that the training of ML models with historical data from IoT devices can bring the capability of forecasting future potential events with high levels of certainty. In support of this, the authors of [191] used 3-year data collected from three locations (Roorkee, Haridwar, and Dehradun in the river Ganga) and monitored 12 physicochemical water parameters: dissolved oxygen (DO), PH, biochemical oxygen demand (BOD), calcium, total coliform, hardness, magnesium, dissolved solids (DS), chlorides, alkalinity, chemical oxygen demand (COD), and temperature. They applied mathematical modelling coupled with ML algorithms to formulate a trend analysis and hence predicted contamination of the three areas from 2020 to 2025. A comparative analysis of the proposed analysis and existing work showed a good match from 2013 to 2021.

While focussing on battery lifetime and longevity of nodes, ref. [192] chose four sites, each with a unique focus for investigation: (1) Ayadat Lake—cyanobacterial evolution; (2) the Allier River—impact on biodiversity by flood; (3) Roffin—the impact of nuclear waste on vegetation and water; and (4) Montoldre—crop development. They built each node to have an assembly of sensors suitable for the purpose for which it was meant and deployed them in their respective areas using a star topology with LoRAWAN as a communication platform to the gateway. The nodes were developed on an STM32 microcontroller and a NoSQL database was included in the structure, while a multi-pipeline architecture was designed for data visualization. Packet retransmission rates varied between 4 and 54% while RSSI values were between −32 dBm to −120 dBm for the node range distance to the gateway. An RF regression model used to identify visibility variables arising from deviations of RSSI values not induced due to the standard log-normal path-loss model, performed poorly by explaining only 49.23% variance. The initial discharge of the battery showed a strong correlation with estimating its lifespan, albeit with approximately 20% precision. Temperature variations at the deployment were not factored in, yet it remains an important basis for scheduling battery maintenance.

In their investigation of oil spillage, the authors of [193] designed a WSN aimed at classifying petroleum products using artificial neural networks. The sensor nodes consisted of an ESP32 microcontroller, LoRa, GPS module, and a pair of 3.7 V/1400 mAh Li-Po batteries charged by solar power. There were three partitions (signal acquisition, energy management, and processing) whose architecture made it possible for data classification to be performed in the embedded structure before forwarding it to the server. Supervised training was done with 7936 samples, with the ANN classifier consisting of three neurons and two hidden layers. The model accurately predicted 83% of samples, while its sensitivity and specificity were 88% and 74%, respectively. Seven out of fifty-four samples were misclassified, and the F score metric value was 86%. This innovative approach seems able to generate good results if applied in pesticide detection.

## 7. Conclusions and Future Directions

Monitoring of water ecosystems is crucial given the increasing global growth rate while the available water resources are declining over time, alongside increasing pollution levels. The release of agricultural runoff into freshwater bodies leading to contamination contributes significantly to water scarcity. Given the hurdles to creating new water sources, the preservation and sustainable management of existing water sources emerge as key strategies in averting prospective crises associated with water scarcity. Future directions in water ecosystem monitoring necessitate the seamless integration of diverse technologies to establish a robust methodology for protecting existing water sources.

Harnessing the power of IoT architectures to enhance transparency and data availability will lead to better-developed models and adaptive management strategies. This necessitates investment in stable and fast connectivity infrastructure to facilitate seamless data exchange among systems. The adoption of 5G connectivity will play a crucial role in enhancing data exchange speeds and improving the accuracy of predictive models.

Future directions involve reducing reliance on cloud-based data management and analysis by developing devices with self-computation capabilities. Fog computing will play a pivotal role in handling large volumes of data locally, hence minimizing energy consumption and optimizing data processing efficiency. Efforts should focus on striking a balance between local processing and cloud support to ensure optimal scalability and performance.

Emphasis should be laid on advancement in sensor technology, prioritizing enhancement of their intelligence to interpret data effectively. This will involve integrating advanced algorithms and machine learning techniques to allow them to autonomously analyse complex environmental data.

Fostering collaboration among various players in the sensor industry is key to successful monitoring. Various organizations are engaged in competitive efforts to establish their technologies as industry standards, a phenomenon with notable implications for interoperability. A move towards the adoption of a unified standard technology and the development of standardized protocols and frameworks across diverse monitoring platforms would be strategic.

## Data Availability

Not applicable.
